# COVID-19 complications in males and females: recent developments

**DOI:** 10.2217/cer-2022-0027

**Published:** 2022-05-05

**Authors:** Rahul Chaturvedi, Briana Lui, Jamie A Aaronson, Robert S White, Jon D Samuels

**Affiliations:** 1 Department of Anesthesiology, Weill Cornell Medicine, 525 East 68th Street, Box 124, New York, NY 10065, USA; 2 Weill Cornell Medical College, Weill Cornell Medicine, 1300 York Avenue, New York, NY 10065, USA

**Keywords:** COVID-19, gender, hospitalization, mortality, SARS-CoV-2, sex

## Abstract

**Aim:**

To provide a comprehensive understanding of the varying effects of SARS-CoV-2 infection based on sex.

**Methods:**

A PubMed search of 470 primary articles was performed, with inclusion based on relevance (sex differences discussed in the target COVID population) and redundancy. PubMed was queried based on title for the keywords “SEX” and “COVID” or “SARS” between 2020 and 2022.

**Results:**

For COVID-19, males have increased risk for infectivity and intensive care unit admission and worse overall outcomes compared with females. Genetic predispositions, sex hormones, immune system responses and non-biological causes all contribute to the disparity in COVID-19 responses between the sexes. COVID-19 sex-related determinants of morbidity and mortality remain unclear.

**Conclusions:**

Male sex is a risk factor for several overall worse outcomes related to COVID-19. Investigating the sex impact of COVID-19 is an important part of understanding the behavior of the disease. Future work is needed to further explore these relationships and optimize the management of COVID-19 patients based on sex.

To date, the rapidly evolving COVID-19 has exceeded 386 million cases globally, 138 million cases within the USA, 5.7 million deaths globally and over 888,000 deaths within the USA [1]. A burgeoning body of literature demonstrates worse outcomes and increased mortality among males with COVID-19 [2]. In retrospective studies, males account for up to 75% of overall COVID-19 deaths [3,4]. Males have three-times the odds of needing intensive care unit (ICU) level of care and have at least a 15% higher risk for mortality than females [5–8]. Males also have higher risk for organ dysfunction such as respiratory failure, acute kidney injury (AKI) and fatal gastrointestinal disease when infected with SARS-CoV-2 [9,10]. Baseline comorbidity differences, such as hypertension, cardiovascular disease and obesity, may play a role; however, when controlling for comorbidities, males still have higher rates of morbidity and mortality; thus, male sex has been identified as a risk factor in some clinical trials [11–13]. Epidemiological data show a similar male bias for poor outcomes for the previous (2002) SARS-CoV infection, as well as for Middle Eastern respiratory syndrome coronavirus (MERS) in 2012 [14].

There is a paucity of studies in the literature examining outcomes in COVID-19 based on sex. In recent studies, less than 5% include sex as an analytical variable [15]. In this review, the authors examine differences in COVID-19 outcomes related to sex, including hospitalization, ICU admission, mortality and cardiac injury. They also discuss factors that may contribute to sex-based discrepancies in COVID-19 morbidity and mortality, within a framework of biological and non-biological causes. The authors report findings from several studies that include sex-disaggregated data primarily filtered by males and females, with intersex and transgender individuals not formally represented. Future research should seek to better understand outcomes for these patient populations as well.

## Outcomes

### Hospitalization & ICU admission

There are several studies that highlight increased hospitalization rates for males with COVID-19, even when correcting for age and other comorbidities [16,17]. Table 1 lists studies exploring differences between males and females with respect to COVID-19, as well their characteristics and the outcomes, primary findings and study limitations. Gomez *et al.* conducted a multicenter, retrospective, cohort study that analyzed differences in rates of hospitalization and ICU admission between males and females from 1 March 2020 to 21 June 2020 within the Rush University Health System. The authors performed a multivariable logistic model controlling for age and several comorbidities and found that males had increased rates of hospitalization (19% vs 13%; p < 0.0002) [16]. Fortunato *et al.* performed a retrospective epidemiological study of hospitalization rates, differences in viral clearance and case fatality rates in a population of patients diagnosed with COVID-19 in the Apulian District of Foggia, Italy, between February and June of 2020 [17]. Males had increased hospitalization rates (45.4%) compared with females (37.9%) (p < 0.01). Viral clearance was higher in females (84.2%) compared with males (79.3%) (p < 0.05), but females had increased length of stay while hospitalized (31.3 ± 14.6) compared with males (26.8 ± 14.4) (p < 0.01). Quaresima *et al.* performed a retrospective study in patients with COVID-19 in Brescia, Italy, in order to better understand the timing of hospitalization and further characterize differences in factors such as age between males and females with COVID-19 [18]. The latency period between symptom onset and hospital admission did not differ between males and females, and the mean age during hospitalization was also similar.

**Table 1.** A summary of available studies on sex and COVID-19 outcomes.

Source (year)	Study design	Country	Sample size (n =)	Defined comparator	COVID-19 outcome	Findings	Limitations	Ref.
Baden *et al.* (2020)	Randomized, observer blinded, placebo-controlled trial	USA	30,420	Males vs females	Vaccine efficacy	Vaccine efficacy was similar in males vs females	Short duration of efficacy	[19]
Bignucolo *et al.* (2021)	Meta-analysis	Global	>30,000 (multiple studies)	Males vs females	Vaccine efficacy	Higher odds of vaccine being efficacious in males compared with females	End point timelines differed between studies	[20]
Fortunato *et al.* (2021)	Retrospective epidemiological	Italy	55,131	Males vs females	Hospitalization rates, viral clearance, mortality	Males had increased hospitalization rates and mortality	Lifestyle, behavioral and social differences not assessed	[17]
Gomez *et al.* (2021)	Retrospective cohort	USA	8108	Males vs females	Hospitalization, ICU admission, endotracheal intubation, mortality	Males had higher rates of hospitalization and ICU transfers	Single health system, early on data from pandemic	[16]
Gupta *et al.* (2020)	Multicenter retrospective cohort	USA	2215	Males vs females	28-day in-hospital mortality	Males had increased odds of 28-day mortality	Baseline risks may have differed between patients of different hospitals	[8]
Hur *et al.* (2020)	Retrospective observational	USA	486	Males vs females	Predictors of intubation in COVID-19 patients	Male sex, age and several other factors predictive of intubation	Intubation criteria differ among providers	[21]
Klang *et al.* (2020)	Retrospective observational	USA	6760	Males vs females	Age, comorbidities (cardiovascular, renal), mortality in those who passed from COVID-19	Males had increased mortality but similar cardiovascular disease profiles; female age was higher than males for those who passed	Urban population, observational study	[22]
Moiseev *et al.* (2020)	Retrospective cohort	Russia	1522	Males vs females	Requirement for mechanical ventilation and mortality rates in the ICU	Under 50 years of age, males had increased ventilation requirements but similar mortality; over 50 years, males had greater mortality rates	Specific subgroup studied (those in ICU on ventilatory support – fairly high overall mortality rate compared with general population)	[23]
Moula *et al.* (2020)	Meta-analysis	Global	8497	Males vs females	Mortality risk	Males had 16% higher mortality risk	Increased heterogeneity in primary end points between papers	[6]
Nepogodiev *et al.* (2020)	Retrospective case control	Global	1128	Males vs females	30-day postoperative mortality in patients with COVID	Males had increased odds of postoperative 30-day mortality	Some patients included based on clinical diagnosis; all types of surgeries included	[7]
Nguyen *et al.* (2021)	Multicenter retrospective observational	USA	308, 010	Males vs females	In-hospital mortality, length of hospital stay, intubation rates	Males had high odds of hospital mortality, length of hospital stay and intubation rates	Vizient database may have inaccurate coding	[24]
Peckham *et al.* (2021)	Meta-analysis	Global	3,111,714	Males vs females	ICU admission and death	Males have three-times odds of requiring ICU admission and increased odds of death	Data on comorbidities such as age, ethnicity and comorbidities not present	[5]
Polack *et al.* (2020)	Placebo-controlled, observer-blinded efficacy trial	Global	43,548	Males vs females	Vaccine efficacy	Vaccine efficacy was similar between males and females	Short follow-up times	[25]
Quaresima *et al.* (2021)	Retrospective	Italy	1000	Males vs females	Hospitalization age, latency between symptom onset and hospitalization	No differences found between age of hospitalization and latency of hospitalization	Reference center hospital for severe illnesses include hematologic, renal and neurological issues	[18]
Raparelli *et al.* (2020)	Retrospective observational	Italy	3517	Males vs females	Comorbidities (ischemic heart disease, chronic kidney disease), dementia, autoimmune diseases) in those who passed from COVID-19	Increased odds for males to experience ischemic heart disease compared with females for those who passed from COVID-19	Only deceased individuals included with unclear baseline comorbidity severity	[26]
Toth-Manikowski *et al.* (2021)	Retrospective cohort	USA	4407	Males vs females	28-day in-hospital mortality, acute kidney injury and respiratory failure within 14 days of ICU admission	Males had increased risk of mortality, severe acute kidney injury and respiratory failure	Immune system response/sex hormone data not ascertained; USA only; acute kidney injury and respiratory failure information only acquired within first 14 days	[10]
Vassilaki *et al.* (2021)	Prospective cohort	Greece	1643	Males vs females	IgG antibody responses to Pfizer vaccine	Females had 1.2-fold higher antibody response	Comorbidities not controlled for; duration of antibody responses followed for short time period	[27]
Xu *et al.* (2020)	Retrospective cohort	China	659	Males vs females	Characteristics of ARDS patients with COVID-19, artificial intelligence model for predicting ARDS	Males had increased risk for ARDS progression	Limited ARDS data, no CT scan imaging for corroboration of diagnosis	[28]

ARDS: Acute respiratory distress syndrome; ICU: Intensive care unit.

COVID-19 progression and rates of ICU admission differ between the sexes. Peckham *et al.* performed an international meta-analysis on 3,111,714 COVID-19 patients between January 2020 and June 2020. Males had almost three-times the odds of requiring ICU admission than females (odds ratio [OR]: 2.84; 95% CI: 2.06–3.92; p = 1.86 × 10^-10^) [5].

### Mortality

Sex-based differences exist in mortality rates for hospitalized patients with severe COVID-19 (Table 1) [6–8,10,16,17,23,29]. Moula *et al.* conducted a global meta-analysis on 26 studies, exploring the impact of sex, age and several other comorbidities on mortality in patients with COVID-19 [6]. Males had a 16% increased risk of mortality compared with females (p < 0.05). Gupta *et al.* conducted a multicenter, retrospective, cohort study in a group of adults with COVID-19 who were admitted to the ICU across 65 sites. Males had increased odds of mortality within the ICU setting (OR: 1.50; 95% CI: 1.19–1.90) [8].

Nepogodiev *et al.* performed a multicenter, retrospective, cohort study across 235 hospitals and 24 countries for patients with COVID-19 infection undergoing surgery (defined as a diagnosis within 7 days prior to or 30 days following surgery) [7]. Males had almost twice the odds of postsurgical 30-day mortality as compared with females (OR: 1.75; 95% CI: 1.28–2.40; p < 0.0001). Moiseev *et al.* investigated mortality differences in different age groups of COVID-19 patients admitted to the ICU for respiratory support [23]. Though males under 50 years of age required increased ventilator support compared with females, mortality rates in this subgroup were similar between the sexes. In patients over 50 years of age, however, males had an increased mortality rate. Despite increased mortality in males with COVID-19, females have a higher chance of long-term COVID-19 manifestations, including fatigue, breathlessness and greater disability after hospitalization with COVID-19 compared with their male counterparts [30].

### Cardiac outcomes

Existing literature suggests that male sex is associated with increased incidence of cardiac complications, though it is not clear if the virus itself has direct deleterious cardiac effects or if males experience worsened secondary systemic consequences due to decreased baseline cardiac function [22,26,31]. Though the clinical correlation was not explored in detail, Ghazizadeh *et al.* found males with COVID-19 to have elevated troponin T levels [26]. Deng *et al.*, however, found that in a retrospective case–control study, elevated cardiac enzymes were likely a sequela of the systemic consequences of illness, as opposed to direct cardiac myocyte damage by the SARS-CoV-2 virus [21]. Raparelli *et al.* performed a case–control analysis of 3517 individuals who had experienced COVID-19-related deaths in Italy. Males had almost two-times the odds of having ischemic heart disease at the time of admission compared with females (OR: 1.76; 95% CI: 1.39–2.23) [22]. In a retrospective observational study, Klang *et al.* analyzed data from patients hospitalized in a large hospital in New York City between March and May 2020 who had died of COVID-19, in order to determine sex differences in age and comorbidities. Although the cardiovascular disease profiles of the genders were similar, mortality was higher in males (women: 18.2% vs men: 20.2%; p = 0.039), and women were on average 5 years older (women: 77.4 +12.7 vs men: 72.4 + 13.0; p < 0.001) [31].

### Pulmonary complications

The current data suggest that males with severe COVID-19 have worse pulmonary complications and a need for more invasive respiratory support [24,28]. Hur *et al.* performed a retrospective observational study among ten hospitals in the Chicago metropolitan area for COVID-19 patients admitted between March 2020 and April 2020. Multivariable logistic analysis showed that males had higher risk for prolonged intubation (OR: 1.69; 95% CI: 1.04–2.77; p = 0.034) [24]. This study, however, was limited in that it included patients in only one region – socioeconomic status, access to healthcare and several other social factors may have impacted the comorbidity burden in this patient population. Nguyen *et al.* performed a larger retrospective study looking at patients diagnosed with COVID-19 between March 2020 and November 2020 across the USA [28]. The authors analyzed outcome measures including length of hospital stay, intubation rates and in-hospital mortality. Males had increased rates of tracheal intubation (21.4% vs 14.6%; p < 0.001), longer hospital stays (9.5 ± 12.5 days vs 7.8 ± 9.8 days; p < 0.001) and increased in-hospital mortality (13.8% vs 10.2%; p < 0.001). Xu *et al.* performed a retrospective cohort study across 11 regions in China and found that male sex was a risk factor for acute respiratory distress syndrome (ARDS) progression in patients with COVID-19 [32].

### Vaccine efficacy

Sex-specific differences in innate and adaptive immunity, and thereby vaccine response, may contribute to differences in ICU admission, mortality and duration of symptoms between the sexes [33,34]. It is important to note, however, that clinical trials tend to exclude females, especially pregnant individuals. For example, data collected from ClinicalTrials.gov show that between January 2020 and January 2021, there were 4420 SARS-CoV-2-related studies, of which only 4% included sex as an analytical variable, and only 18% reported sex-disaggregated data [19].

Baden *et al.* conducted a phase III randomized, observer-blinded, placebo-controlled trial across 99 centers in the USA comparing vaccine efficacy of the mRNA-1273 (Moderna) vaccine versus placebo, with the primary end point being the prevention of COVID-19 at least 14 days after the second dose in those not previously infected with SARS-CoV-2 [25]. The efficacy of the vaccine in males (95.4; 95% CI: 87.4–98.3) was not statistically different from that than in females (93.1; 95% CI: 85.2–96.8). Polack *et al.* performed a similar trial for the BNT162b2 (Pfizer) vaccine and included data for the 14 weeks following the second dose [35]. The efficacy of the vaccine in males (96.4; 95% CI: 88.9–99.3) was similar to that in females (93.7; 95% CI: 84.7–98). The US FDA briefing document for the Ad26.COV2.S (Johnson & Johnson) vaccine showed that its efficacy measured 14 days after the second dose was higher in males (68.8; 95% CI: 60.1–75.9) than in females (63.4; 95% CI: 53.1–71.7) [27]. Males were also found to have higher vaccine efficacy rates (69.8; 95% CI: 58.9–78.2) when the primary end point for COVID disease was extended to 28 days than were females (60.3; 95% CI: 46.0–71.2), though the differences were not statistically significant. Vassilaki *et al.* performed a prospective cohort study in which they compared the anti-SARS-CoV-2 spike receptor-binding domain (RBC) IgG antibody levels between males and females 20–30 days after the second BNT162b2 vaccine [20]. Females had a 1.2-mean-fold (p = 0.000003) higher antibody response. Bignucolo *et al.* performed a meta-analysis comparing vaccine efficacy between males and females, including data from Pfizer, Moderna, Gamaleja and Johnson & Johnson trials [36]. The conducted analysis found that vaccination was more effective in preventing COVID-19 in males than in females (OR: 0.67; 95% CI: 0.48–0.94) [36]. Of note, previous data from influenza trials demonstrate that females can mount similar immunological responses to half the vaccine dose received by men [37].

## Potential explanations: biological factors

### Genetic expression differences

The role of ACE2 continues to be central in understanding COVID-19 disease. The gene responsible for ACE2 expression (*Xp22.2*) lies in the X chromosome. Females have two copies and thereby double the amount of ACE2, which may compensate for SARS-CoV-2-mediated downregulation of ACE2 cell surface expression [38]. Females also have X chromosome mosaicism – different cells will express a slightly different allelic variation of the gene, with some being more resistant to SARS-CoV-2 virus binding than others [39].

The ACE2 receptor has also been shown to be expressed preferentially in the small airway tissue in male smokers and patients with chronic obstructive pulmonary disease (COPD) (Table 2) [40]. Androgen receptors serve as promoters for the transcription of *TMPRSS2*, an endogenous gene that participates in normal prostatic function. In high androgen states, TMPRSS2 proteolytic activity is enhanced, cleaving the SARS-CoV-2 spike protein and increasing host susceptibility to SARS-CoV-2 infiltration [41]. The Y chromosome plays a role in the expression pattern of CD4^+^ T cells, the response of macrophages and the number of natural killer T cells, most likely contributing to the differing adaptive and innate immune responses males and females have in response to SARS-CoV-2 [42]. Li *et al.* showed that the testis may be infected by SARS-CoV-2 and found that there is increased expression of ACE2 in Sertoli and endothelial cells of the testis [42,43].

**Table 2.** A summary of studies exploring mechanisms for differing COVID-19 outcomes by sex.

Source (year)	Study design	Country	Sample size (n =)	Defined comparator	COVID-19 outcome	Findings	Limitations	Ref.
Dhindsa *et al.* (2021)	Retrospective cohort	USA	152	Hormone levels	Cytokine concentration and disease severity	Lower testosterone associated with increased severity of disease	Did not measure free testosterone	[44]
Leung *et al.* (2020)	Cross-sectional	Global	16 datasets (analyzed separately)	COPD vs control, males vs females	ACE2 expression in those with COPD vs controls, also controlling for sex	Increased ACE2 expression in males with COPD	Bronchodilators/other medications could be impacting ACE2 expression	[39]
Li *et al.* (2020)	Retrospective cohort	Global	31	Males vs females	Tissue expression ACE2	Testis, small intestine, kidneys, heart, thyroid and adipose tissue had highest ACE2 expression	Small sample size, no protein expression data	[43]
Montopoli *et al.* (2020)	Retrospective cohort	Italy	9280	Male prostate cancer with androgen deprivation therapy vs without	Infectivity rates	Those with androgen deprivation therapy had decreased infectivity rates	Cancer population specifically	[45]
Solomou *et al.* (2020)	Cross-sectional	Cyprus	1642	Males vs females	Depression, anxiety and precautionary compliance related to COVID-19	Males demonstrated decreased COVID-19 precautionary compliance	Response bias (survey-based), no data for those >60	[46]
Takahashi *et al.* (2020)	Experimental	USA	98	Males vs females	T-cell differentiation, disease severity	Males had decreased T-cell differentiation compared with females	Possible confounding variables when comparing healthy controls with those with disease not accounted for when matching	[47]
Yan *et al.* (2020)	Retrospective cohort	China	1004	Males vs females	In-hospital mortality, neutrophil to lymphocyte ratio	Males had an increased neutrophil to lymphocyte ratio, which corresponded to increased mortality	Comorbidities and medications affect all-cause mortality	[29]
Zeng *et al.* (2020)	Cross-sectional	China	331	Males vs females	IgG response based on disease severity	Females mounted an increased IgG response in severe disease but a similar response in mild to moderate cases	Small sample size, single hospital	[48]

COPD: Chronic obstructive pulmonary disease.

### Sex hormones

Hormone variability of androgens, estrogen and progesterone may play a role in the differing morbidity and mortality for COVID-19 between the sexes. Many negative sequelae of SARS-CoV-2 infection are related to the excess release of inflammatory factors, such as interleukins and TNF-α [49]. Peri-ovulatory estrogen has been shown to decrease interleukin release (predominantly IL-6 and IL-8) as well as TNF-α, with decreased estrogen levels leading to increased inflammatory mediators [50]. Postmenopausal women, therefore, have both elevated interleukin (IL-1, IL-6) and TNF-α levels, which decrease in response to supplemental hormone replacement. Estrogen disrupts glycosylation and limits cytokine release, decreasing the penetration of the SARS-CoV-2 virus into the cell and limiting inflammation [51,52].

Increased androgenic activity strengthens SARS-CoV-2 binding to ACE2 by serving as a promoter for the *TMPRSS2* gene and increases infectivity [53]. Studies have demonstrated that a significant portion of COVID-19 patients admitted to the ICU had androgenetic alopecia, suggesting that excess androgen is a poor prognostic indicator and increases susceptibility to escalation of care [45]. Montopoli *et al.* found that those receiving androgen deprivation therapy (ADT) showed decreased infectivity with COVID-19 (Table 2) [44]. Pagano *et al.* found that males with mild to no signs of ARDS tend to have significantly higher testosterone levels than those with moderate to severe ARDS [54].

There is evidence to suggest that increased androgenic activity leads to increased COVID-19 infectivity, morbidity and mortality. A recent Journal of the American Medical Association study found an inverse relationship between testosterone and various inflammatory factors, such as IL-6, C-reactive protein (CRP) and other inflammatory factors (Table 2) [55]. Paradoxically, subnormal testosterone level was associated with increased disease severity and pro-inflammatory states [55]. Agents such as bicalutamide and enzalutamide (anti-androgen agents), as well as camostat (TMPRSS2 inhibitor) are currently being studied and may prove useful in the fight against COVID-19 [56].

### Immune system

#### Innate

An increased release of several inflammatory biomarkers, such as IL-6, IL-2, IL-8 and IL-10, can lead to worsening edema and ventilation in the lung parenchyma [48]. Mouse models have shown elevated levels of IL-2, TNF-α, CCL14, CCL23, IL-7, IL-16 and IL-18 in male lines [47]. Neutrophil to lymphocyte ratio (NLR) is positively correlated with increased severity of COVID-19 [29]. Yan *et al.* performed a retrospective, single-center study showing that males had an NLR greater than 11.75 and a decreased survival rate [29]. Another component important in the innate immune defense system is the presence of toll-like receptors (TLRs), which are responsible for upregulating type 1 interferon (IFN); studies have found increased expression of these receptors in females compared with males [29]. Lau *et al.* examined differences in inflammatory markers between males and females with COVID-19 at Massachusetts General Hospital between March and April of 2020. Both initial and peak CRP levels were higher in males than in females after adjusting for several parameters, with male sex being a positive modifier for the association of peak CRP levels with death and ICU admission [57].

#### Adaptive

Analysis of the serology from patients with mild symptomatic COVID-19 and those who are recovering from the disease does not show a significant difference in the concentration of IgG between males and females [58]. However, for those patients with severe symptoms, female patients tend to have both elevated levels of IgG antibodies against SARS-CoV-2 and stronger antibody response during the earlier phases of the disease process [58]. Takahashi *et al.* explored how T-cell differentiation differed between the sexes. The group found that, overall, females tended to have increased terminally differentiated T cells at baseline. When comparing males with severe disease versus more mild cases, those with worsened disease had both lower proportions of activated T cells and terminally differentiated T cells. When comparing women with severe illness versus more mild cases, the study found similar levels of T-cell response; however, when comparing both female groups (less severe and more severe cases), increased innate immune response was associated with worsened outcomes. The study pointed to the idea that vaccine and pharmacological therapies should target T cells and the adaptive immune system, as it plays a large role in effectively overcoming COVID-19 [59]. Other studies have found that healthy females tended to have increased CD4^+^:CD8^+^ T-cell ratios, cytotoxic T cells and B cells compared with males [46].

## Potential explanations: non-biological factors

A higher proportion of men have been shown to engage in deleterious health behaviors, namely smoking and alcohol consumption [60]. Smoking has been associated with increased expression of ACE2 receptors and may thereby impact SARS-CoV-2 entry into cells [61]. Solomou *et al.* showed that males exhibit decreased compliance with precautionary measures to limit the spread of COVID-19, such as social distancing and masking, when compared with females [62]. It is interesting that certain professional groups, such as frontline healthcare workers, are disproportionally female (e.g., nurses, health technicians, community health workers), which would increase exposure and risk of infection [12]. While the overall incidence of COVID-19 among the general population lies around 2%, current data indicate that the incidence among healthcare professionals is closer to 5–6%; females tend to be more affected (53.5%) than males (46.5%), though the data are not statistically significant, with nurses constituting the majority of infected cases [63,64].

Sexual health has also been impacted significantly by the COVID-19 pandemic, and there are data to suggest that COVID-19 can be transmitted sexually [65]. Kumar *et al.* found that men who have sex with men may have similar rates of engagement in sexual activity as pre-pandemic levels, though sexually transmitted infection and HIV testing rates, along with condom use, have been declining, possibly due to access. Research on sex workers, a predominantly female-driven industry, has shown that in-person sexual work and activity have declined as anxiety regarding COVID-19 has increased. The study also found that the data regarding women's sexual behavior and mental health during COVID-19 have been scarce; while that may be due in part to search criteria, it may also partially be due to the ‘male-as-norm bias’ that pervades health research [65].

## Conclusion & future perspective

The current COVID-19 pandemic has led to millions of deaths globally and has disproportionally impacted males. Several factors explain the increased morbidity and mortality that males experience when infected with COVID-19 – genetic predisposition, hormone effects, immune system responses and non-biological causes, such as smoking and alcohol consumption. Future studies are needed, especially in the form of clinical trials, that involve sex as a variable for analysis in order to better guide the development of tailored therapy against SARS-CoV-2.

**Executive summary**

- There is a COVID-19 pandemic. Although most infected patients have mild symptoms, approximately 20% have severe disease, including pneumonia, respiratory failure, septic shock and multisystem organ failure.
- There is a growing body of literature highlighting worse outcomes and increased mortality among males with severe COVID-19.
- Several genetic factors, such as the role of ACE2, continue to be central to better understanding the sex differences.
- Hormone variability of androgens, estrogen and progesterone may play a role in the differing morbidity and mortality for COVID-19 between the sexes.
- Both the innate and adaptive immune system responses to COVID-19 vary by sex and can contribute to worse outcomes in males.
- Several non-biological differences between the sexes, such as smoking and alcohol consumption, may play a crucial role in the differences in morbidity and mortality between males and females.
- Improved understanding of these various sex differences can help lead to more targeted pharmacological therapies and vaccine production.
